# Estimating oxygen distribution from vasculature in three-dimensional tumour tissue

**DOI:** 10.1098/rsif.2016.0070

**Published:** 2016-03

**Authors:** David Robert Grimes, Pavitra Kannan, Daniel R. Warren, Bostjan Markelc, Russell Bates, Ruth Muschel, Mike Partridge

**Affiliations:** 1 Cancer Research UK/MRC Oxford Institute for Radiation Oncology, Gray Laboratory, University of Oxford, Old Road Campus Research Building, Off Roosevelt Drive, Oxford OX3 7DQ, UK; 2 Engineering Sciences, University of Oxford, Oxford OX1 3PJ, UK

**Keywords:** oxygen, cancer, modelling, hypoxia, radiotherapy

## Abstract

Regions of tissue which are well oxygenated respond better to radiotherapy than hypoxic regions by up to a factor of three. If these volumes could be accurately estimated, then it might be possible to selectively boost dose to radio-resistant regions, a concept known as dose-painting. While imaging modalities such as ^18^F-fluoromisonidazole positron emission tomography (PET) allow identification of hypoxic regions, they are intrinsically limited by the physics of such systems to the millimetre domain, whereas tumour oxygenation is known to vary over a micrometre scale. Mathematical modelling of microscopic tumour oxygen distribution therefore has the potential to complement and enhance macroscopic information derived from PET. In this work, we develop a general method of estimating oxygen distribution in three dimensions from a source vessel map. The method is applied analytically to line sources and quasi-linear idealized line source maps, and also applied to full three-dimensional vessel distributions through a kernel method and compared with oxygen distribution in tumour sections. The model outlined is flexible and stable, and can readily be applied to estimating likely microscopic oxygen distribution from any source geometry. We also investigate the problem of reconstructing three-dimensional oxygen maps from histological and confocal two-dimensional sections, concluding that two-dimensional histological sections are generally inadequate representations of the three-dimensional oxygen distribution.

## 1. Introduction

The fact that oxygen status of tissue plays an important role in how a patient responds to treatment has been known since the pioneering work of Gray *et al.* in the 1950s [1], who documented the fact that well-oxygenated tissue responds better to radiotherapy by a factor of 2.7–3 relative to regions with extensive hypoxia. This treatment-boosting effect is usually ascribed to the oxygen fixation hypothesis, which gives a chemical rationale for what is observed. In its simplest form, this hypothesis states that in presence of oxygen, DNA damage by ionizing radiation can be ‘fixed’, and rendered difficult or impossible for the cell to repair. Conversely, when oxygen levels are low, this mechanism does not occur [2]. The relative boosting effect of oxygen on cell-kill is quantified by the oxygen enhancement ratio (OER), which is of fundamental importance in radiotherapy [3], the mechanism of action of which has recently been explored [4].

The extent of tumour hypoxia can be a useful prognostic marker, and is currently being researched as a driver for dose-painting in radiotherapy [5,6]. In dose-painting, hypoxic regions of a tumour could be given an increased radiation dose to mitigate the inherent radioresistance of a low oxygen environment. While imaging modalities such as positron emission tomography (PET) can use hypoxia binding tracers such as ^18^F-fluoromisonidazole [7] to allow the non-invasive estimation of hypoxia *in vivo*, the intrinsic spatial resolution remains limited by the physics of PET to the millimetre domain, whereas oxygen status can vary markedly over a micrometre scale owing to the limited oxygen diffusion distances (of the order of 100–200 µm [8–11]) in respiring tissue. To bridge the divide between these length-scales, mathematical modelling of the microscopic distribution of oxygen must be employed [12].

The classic Krogh model of a cylinder is a simple and often-used model for vessel oxygenation [10,12,13]. It assumes cylindrical diffusion of oxygen through a respiring tissue perpendicular to the vessel wall. Because it was originally proposed nearly a century ago, this model and variations on it have become practically ubiquitous and have been used for various applications, such as estimating oxygen diffusion in tissue from single vessels [10], investigating drug delivery [14] and recently in determining the washout of inert solutes from tissue [15]. The model is simple to apply and comprehend, but relies on several assumptions, many of which may be unrealistic; specifically, it assumes no axial or azimuthal diffusion, homogeneous distribution of blood vessels and that all vessels are straight with even flow [13]. Many extensions of Krogh's initial work have been proposed with varying levels of mathematical complexity to account for various weaknesses in the model [16–18], yet even the basic model still has ready applicability, particularly when diffusion is considered from a unidirectional source [13].

Another complication that arises in oxygen modelling is the fact that in tumour tissue, the supplying vasculature can be highly contorted, poorly perfused and chaotic [12,19]—this can result in lower venous pressure than in normal well-perfused vessels, such as 20–30 mmHg versus the typical venous pressure of 40 mmHg. The chaotic situation has been long observed [20,21], with disordered vasculature a common problem in solid tumours. While the simple Krogh model has had numerous applications, it is in general not suitable for complex vasculature. Many more complex models of vessel formation arise in the field of applied mathematics, generally using partial differential equations (PDEs) solved with numerical finite difference methods or cellular automaton models; these tend to incorporate estimation terms for oxygen distribution and vessel flow [22,23]. Others have used different approaches, such as that of Secomb *et al.,* who took a Green's function approach to model oxygen distribution using the impulse response of the system [24]. Many of these models are highly sophisticated and of considerable benefit in predicting characteristic tumour behaviour such as angiogenesis and invasion. Depending on complexity, such models are generally confined to a two-dimensional lattice of points and are not typically designed to produce full three-dimensional oxygen maps, though many can be adapted to factor this in at increased computational expense involved in solving numerical equations in extra dimensions. When dealing with the complex nature of tumour vasculature, the three-dimensional distributions appear to be of fundamental importance, and this avenue is in need of deeper investigation.

As microscopy continues to improve, it has become technically feasible to obtain more detailed vessel maps and perfusion data at different resolutions. A simple stable model for estimating the likely oxygen distributions across these length-scales in all three-dimensions would be of considerable interest in ascertaining the nature of hypoxia. In this work, we will examine oxygen distribution in three dimensions, considering analytical line source and kernel-based formulations. In these models, oxygen diffusion is not confined to a perpendicular direction, and axial diffusion is intrinsically considered. Line sources can be thought of as a linear array of point sources, radiating spherically in space. They are especially useful for modelling extended sources, such as traffic flow or electromagnetic radiation incident upon a patient and reflection from long fluorescent tubes used in ultraviolet phototherapy [25–27], but to the authors' knowledge have not been used with vessel sections before. We establish a model for a line source vessel and also a process to kernelize and normalize this for any arbitrary vessel geometry, including the chaotic vasculature often encountered in tumours. The model derived here is simple to implement, completely analytical, always numerically stable and readily accommodating of different length-scales and vessel resolutions. This model will be applied to tumour sections of various microvessel density as generated by Secomb *et al.* and also vessel maps derived from high-resolution confocal imaging of MC-38 mouse tumours created specifically for this work. We also investigate whether two-dimensional histological slices might be considered representative of a three-dimensional vessel network, by contrasting three-dimensional oxygen maps to simulated two-dimensional analogues.

## 2. Model derivation

**Figure 1.**
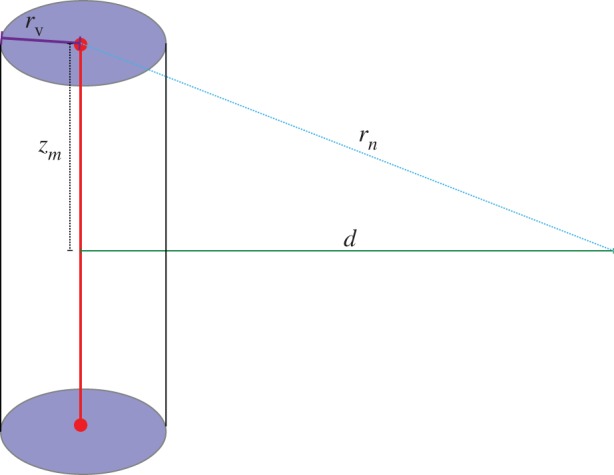
Line source model of a vessel with radius *r*_v_; at a perpendicular distance *d* from the vessel, contributions along *z* span −*z_m_* ≤ *z* ≤ *z_m_*.

We consider a vessel as an extended line of oxygen point sources along its axis as depicted in figure 1. For an infinitesimal point on this axis d*z*, oxygen diffuses as a point source. Oxygen distribution can be written as a reaction–diffusion equation in spherical coordinates, and under the assumption that oxygen distribution is steady state and oxygen consumption rate *a* is approximately uniform, then the oxygen distribution from a spherical source can be derived analytically subject to appropriate boundary conditions, specifically that oxygen can diffuse a distance of *r_n_* from the source until it is fully depleted. A full mathematical treatment for spherical oxygen diffusion has been previously derived for isotropic oxygen transport in tumour spheroids [11]. A similar approach can be employed to describe spherical oxygen diffusion from a point source with appropriate boundary conditions. Full mathematical details are omitted here for brevity but given in electronic supplementary material, appendix S1(a). We may state that for a line source of such spherical points, the infinitesimal contribution from a unit length d*z* is given by
2.1
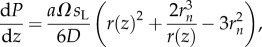
where *D* is the oxygen diffusion constant (approx. 2 × 10^−9^ m^2^ s^−1^ [10,11] in tumour tissue), *a* is the oxygen consumption rate (volume of oxygen per unit mass per unit time) and *r* is radial vector from an element d*z* a perpendicular distance *d* from the vessel centre, so that 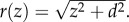 The diffusion distance is *r_n_*, representing the maximum distance oxygen can travel from the vessel centre before being depleted by the respiring tissue. The constant 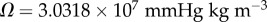 arises from Henry's law [11], and *s*_L_ is defined as oxygen contribution per unit source length. As oxygen from a point source falls off rapidly with increasing distance, we further assume that sources are approximately independent of one another along the length of the vessel. Equation (2.1) may be expressed in terms of *z* and integrated between the limits *z_m_* and −*z_m_*. Writing 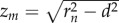 and solving yields
2.2
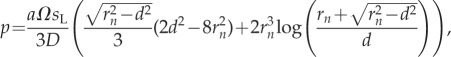
for *r* ≤ *r_n_*, with *p* = 0 beyond the diffusion limit *r_n_*. This identity yields a line source model for an extended vessel, whose effective length at a radial distance *d* is the sum of contributions along the contributing length 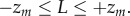 Unlike the Krogh model, the line-source model does not assume that oxygen diffusion is solely perpendicular to the vessel wall, instead considering the situation where oxygen diffuses from the vessel in all directions. For the sake of simplicity, we will initially not consider flow or the potential for diminishing partial pressure along the vessel length, assuming that pressure decreases are small enough to be negligible along small vessel segments, though this can be readily factored in and is discussed later. One limitation of this method is that the oxygen consumption per unit length term *s*_L_ is degenerate with the oxygen consumption rate *a*. Thus, given only an experimentally determined diffusion limit *r_n_*, one could determine the product of *a* and *s*_L_ rather than uniquely determining both. However, there are methods for determining the oxygen consumption rate [11] that could allow separation of these two values under some experimental conditions.

We define *p* = *p*_o_ at the vessel wall (*d* = *r*_v_), and from this show the effective diffusion distance *r_f_* is related to the vessel radius in a manner analogous to the effect of vessel radius in the Krogh model [10,12]. This reflects that idea that subject to the same boundary conditions, larger vessels diffuse more oxygen that can travel further and vice versa. If we initially take the radius of a capillary as *r*_o_ = 5 µm, we can define two distinct extremes that correspond to well-perfused and poorly perfused situations.

(1) *Well-perfused condition*: in this situation, the vasculature is well perfused, and the vessel wall is at typical venous pressure of approximately 40 mmHg. The diffusion distance through the tissue is taken from the literature to be approximately 150 µm [8–10].
(2) *Poorly perfused condition*: as poorly perfused vessels are often a consequence of chaotic tumour vasculature, we also model a condition where oxygen partial pressure at the vessel wall can be as low as 20 mmHg [19]. To illustrate this extreme further, we also assume that in this tissue, the oxygen consumption rate is high which acts to reduce the diffusion distance [11,12] and so in this situation, the diffusion distance from a capillary of *r*_o_ = 5 µm is reduced to *r_n_* = 100 µm.

Thus, in this work, the optimum scenario for high oxygen diffusion (long diffusion distance, high perfusion) and the worst-case scenario (short diffusion distance, low perfusion) can be directly contrasted as two extremes. To scale the line source and kernel with respect to the vessel radius is both the well-perfused and poorly perfused situation, we first calculate the respective values of *as*_L_ by
2.3
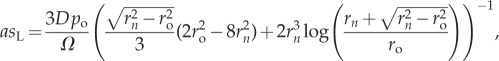
where *p*_o_ is the oxygen partial pressure at the vessel wall. For the well-perfused situation here, this yields 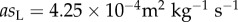 and for the poorly perfused situation, 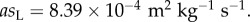 The scaled diffusion distance *r_f_* from a vessel of radius *r*_v_ in either situation is thus given by solving
2.4
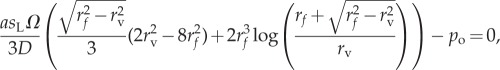
for *r_f_*. Table 1 illustrates the scaled diffusion distances for a wide range of *r*_v_ under the well and poorly perfused assumptions.

**Table 1.** Effective diffusion distances for differing vessel radii in the line source model. Values in italics indicate the default situation of a vessel with radius 5 µm having diffusion distance *r*_*n*_ — other entries are scaled from this. See §2 for details.

vessel radius *r*_v_ (µm)	diffusion limit *r_f_* (high perfusion) (µm)	diffusion limit *r_f_* (low perfusion) (µm)
1	130.15	85.24
2	137.44	90.57
3	142.51	94.33
4	146.85	97.37
*5*	*150*	*100*
10	162.97	110.13
20	181.01	124.88
30	195.32	137.09
40	207.99	148.23
50	219.72	158.79

### 2.1. Kernel model

**Figure 2.**
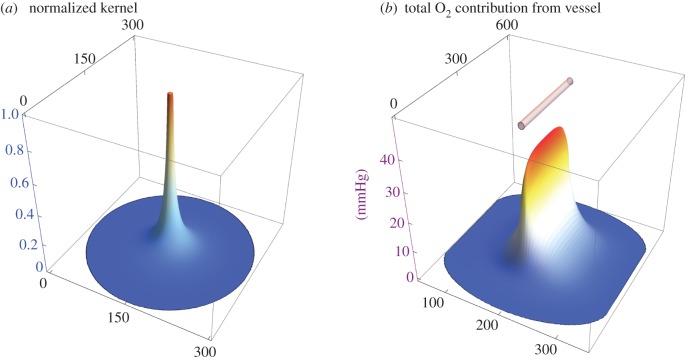
(*a*) The kernel produced for a point source with 150 µm diffusion distance normalized to unity. (*b*) The resultant oxygen map taken through the central plane of the 200 µm vessel shown in the top of the figure, with an external partial pressure of 40 mmHg. The *x-* and *y*-axes in both parts of the figure depict displacement in micrometres.

The model presented in equation (2.2) is analytical and suitable for any long approximately linear vessel segment. However, real tumour vasculature tends to be chaotic, so a linear approximation is often of limited use. To circumvent this, we may use the underlying theory to yield a suitable discrete approach along any vessel length and shape. For a known vessel geometry, we take each vessel segment and subdivide it into *N* discrete elements, placing a predefined spherical kernel at each position *x_N_*, *y_N_*, *z_N_* along the vessel, numerically summing the contributions from each. This summation can then be normalized to typical partial pressure at the vessel wall. These kernels are also scalable, so effective diffusion distance from a vessel is a function of vessel radius, analogous to the Krogh model. For each vessel segment of length *L*, the radius *r*_v_ is estimated, and from this the scaled oxygen diffusion distance *r_f_* can be obtained as per the method outlined in the previous section. The unscaled kernel is generated for that vessel segment by using the distribution described by equation (2.1), yielding
2.5
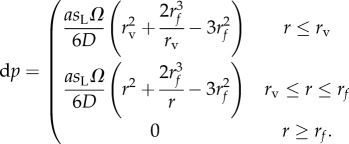
The kernel is then placed at equidistant discrete points along the vessel segment and numerically summed to produce an unscaled oxygen contribution map from this segment. In this work, the discrete spacing was set to 1 µm, and a robust method for finding precise kernel locations with any chosen spacing is shown in the mathematical appendix S1(b) in the electronic supplementary material. Finally, the oxygen map for this vessel is normalized to the expected pressure at a position *W* on the vessel wall, a perpendicular distance *r_f_* from the vessel centre point *C*. As the vessels can be orientated any direction in space, a robust method for accurately calculating the coordinates of *W* must be employed for an accurate estimate; a concise and powerful method for doing this is also outlined in mathematical appendix S1(c) in the electronic supplementary material. To normalize the vessel, first the length *L* of the segment is considered; if the segment length *L* is sufficiently great that the point *W* gets all contributions from the vessel, then the partial pressure at this distance is simply *P_w_* = *p*_o_. However, if the segment is smaller than this limit, then the partial pressure for normalization will be lower as there is less contribution from the vessel, as can be seen from manipulation of equation (2.2). If the segment is sufficiently long (
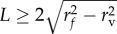
) then the normalization constant *P_w_* is simply *p*_o_. For small vessel segments (
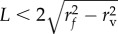
), the partial pressure at the vessel wall is less than *p*_o_, and is given by
2.6
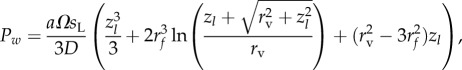
where *z_l_* = *L*/2. The scaling factor for normalization can be simply calculated from this; if the unscaled partial pressure at *W* is *U_w_*, then the scaling factor for the entire segment is given by
2.7
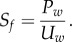
Finally, the entire unscaled vessel segment is scaled by *S_f_* to produce the oxygen map from the segment. This can be independently run on any desired number of segments in three-dimensional space, and the results summed to produce a full three-dimensional oxygen map in a given volume. This process is illustrated in figure 2 for a single 200 µm long vessel segment of radius *r*_v_ = 5 µm under the assumption of high perfusion, with the oxygen map taken through the vessel centre.

## 3. Experimental method

### 3.1. Generation of three-dimensional oxygen maps from existing data

**Figure 3.**
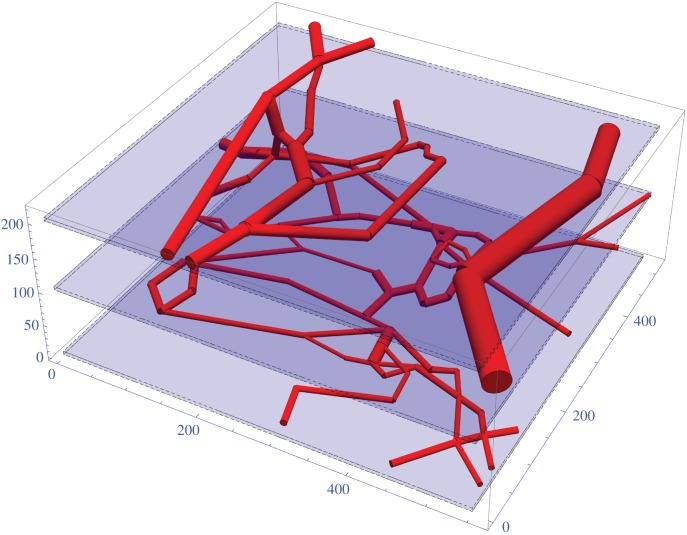
Rendered vessel maps for a 104 segment 555 × 525 × 215 rat carcinoma. Slice planes are indicated by blue regions and discussed in text. All axes in micrometres.

Vessel maps from rat tumours have previously been derived and made available by Secomb *et al.* [20,21]. A vessel dense segment of rat carcinoma is illustrated in figure 3. The kernel model was applied to the rat carcinoma in this work under both highly and poorly perfused assumptions. The model is also applied to a simpler high-density rat tumour in the electronic supplementary material, S1(d), omitted here for brevity.

### 3.2. Generation of three-dimensional oxygen maps from vessels of a mouse tumour

The kernel model was also applied to a murine tumour with perfusion data, so only functional vessels were considered—colorectal cell line MC38 was cultured in Dulbecco's modified Eagle's medium supplemented with 10% fetal bovine serum and 1% penicillin/streptomycin. Cells were grown at 37°C in an incubator containing 5% CO_2_. To obtain three-dimensional oxygen maps from vessels of a mouse tumour, MC38 cells (1 × 10^6^ cells in 100 µl phosphate-buffered saline) were injected subcutaneously onto the right flank of an eight-week-old female C57/Bl6 mouse (Charles River). When the tumour reached 40 mm^3^ in volume, markers for perfused vessels (anti-mouse CD31 antibody FITC-labelled, 50 µg in 100 µl, BioLegend) and tissue (Hoechst 33342, 1 mg ml^−1^ in saline, 50 µl, Sigma Aldrich) were injected into the lateral tail vein 10 min and 1 min prior to culling. The tumour was excised, cut in half and placed for imaging in a humidified chamber slide with a cover glass-like bottom. Perfused vessels and tissue were imaged using a Zeiss LSM710 bi-photon microscope (Carl Zeiss AG), connected to a Mai-Tai tuneable laser. Tissue was excited with a laser wavelength of 800 nm and the emitted light was collected on a non-descanned detector through a 457–487 nm bandpass filter for Hoechst and on a gallium arsenide phosphide detector through a 500–550 nm bandpass for FITC. A 20× water immersion objective with NA of 1.0 was used to acquire a Z-stack of a random region in the centre of tumour tissue. The size of the acquired Z-stack was 425 × 425 × 272 µm with a pixel size of 0.415 µm in *X* and *Y* and a *Z* step of 2 µm.

**Figure 4.**
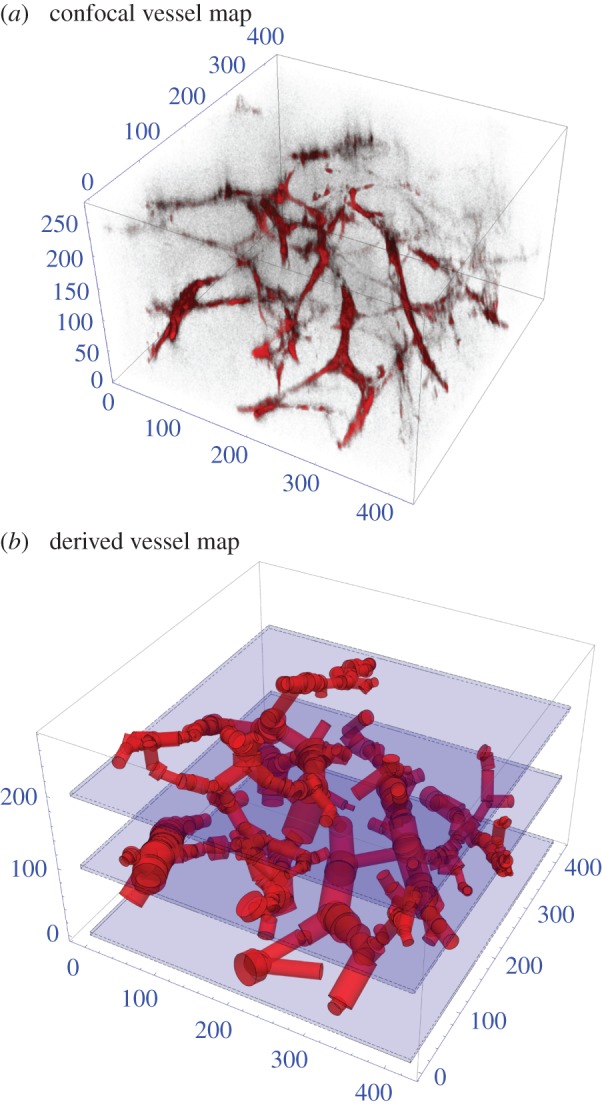
(*a*) Confocal imaging of the 357 vessel sections in a 425 × 425 × 272 µm^3^ mouse tumour. (*b*) Vessel segmented through detection algorithm for this section. All axes in micrometres.

With the perfused vessels stained, segmentation of vessels was performed using a multiscale Hessian-based vesselness filter inspired by analysis of the stress tensor of solid mechanics [28]. By analysing the second-order structure of an image at various scales, we can determine regions that are part of tubular structures such vessels. The likelihood map generated by this approach was binarized using an Markov random field (MRF)-based approach to encourage contiguous regions [29]. We skeletonize the vessel segmentation using an iterative thinning approach. Points on the skeleton can then be characterized by how many neighbours they have. Branching points will have greater than two neighbours, whereas endpoints will have only one neighbour. The branch thickness was determined using a distance map transform on the segmentation. The vessel network can then be approximated by straight branches connecting the branch points, whose thickness is calculated from the extracted skeleton and the distance map, a process illustrated in figure 4.

### 3.3. Validation and comparison with two-dimensional histology

#### 3.3.1. EF5-stained tumour sections

Obtaining accurate oxygen distributions at the micrometre scale in tumour tissue remains technically challenging, with direct validation not yet feasible. We were able to ascertain oxygen maps of larger slices, achieved by growing full MC-38 mouse tumours and staining these with the hypoxia marker EF5. EF5 has a specific binding range, manifesting at oxygen partial pressures of between 0.8 and 10 mmHg [30]. The mouse models used for validation were the same as used for the generation of three-dimensional oxygen maps, following the protocol described in §3.2. Tumours were grown in five eight-week-old female C57/Bl6 mice (Charles River). When the tumour's volume reached 40–60 mm^3^ (calculated as length × width × height × *π*/6), markers for hypoxia (EF5, 10 mM, intraperitoneal) and perfused vessels (anti-mouse CD31 antibody FITC-labelled, 50 µg in 100 µl intravenous, BioLegend) were injected 2 h and 10 min prior to culling. Tumours were excised, embedded in freezing-medium and snap-frozen in liquid nitrogen. Sections (10 µm thick) were cut from each tumour and stained for hypoxia using anti-EF5 antibody (obtained from Dr Cameron Koch) and for nuclei (Hoechst 33342, 1 mg ml^−1^) according to published protocols [30]. An adjacent slide was also taken but not stained with EF5, and functioned as a control to ensure that detected EF5 in the active slice was not an error artefact. Immunofluorescently stained slides were imaged with an epifluorescence microscope Nikon Eclipse Ti-E (Nikon) with the following filter pairs: DAPI (Ex: 350/50 nm, Em: 460/50 nm), PE (Ex: 560/40 nm, Em: 630/75 nm) and Cy5 (Ex: 620/60 nm, Em: 700/75 nm). A tile-scan of the entire tumour section was acquired with NIS Elements software (Nikon) with a 16-bit sCMOS camera (Hamamatsu Orca-Flash 4.0). Files were stored as 16-bit per channel TIFFs and analysed off-line. Total tumour area of the sections ranged from 12.35 to 35.5 mm^2^.

**Figure 5.**
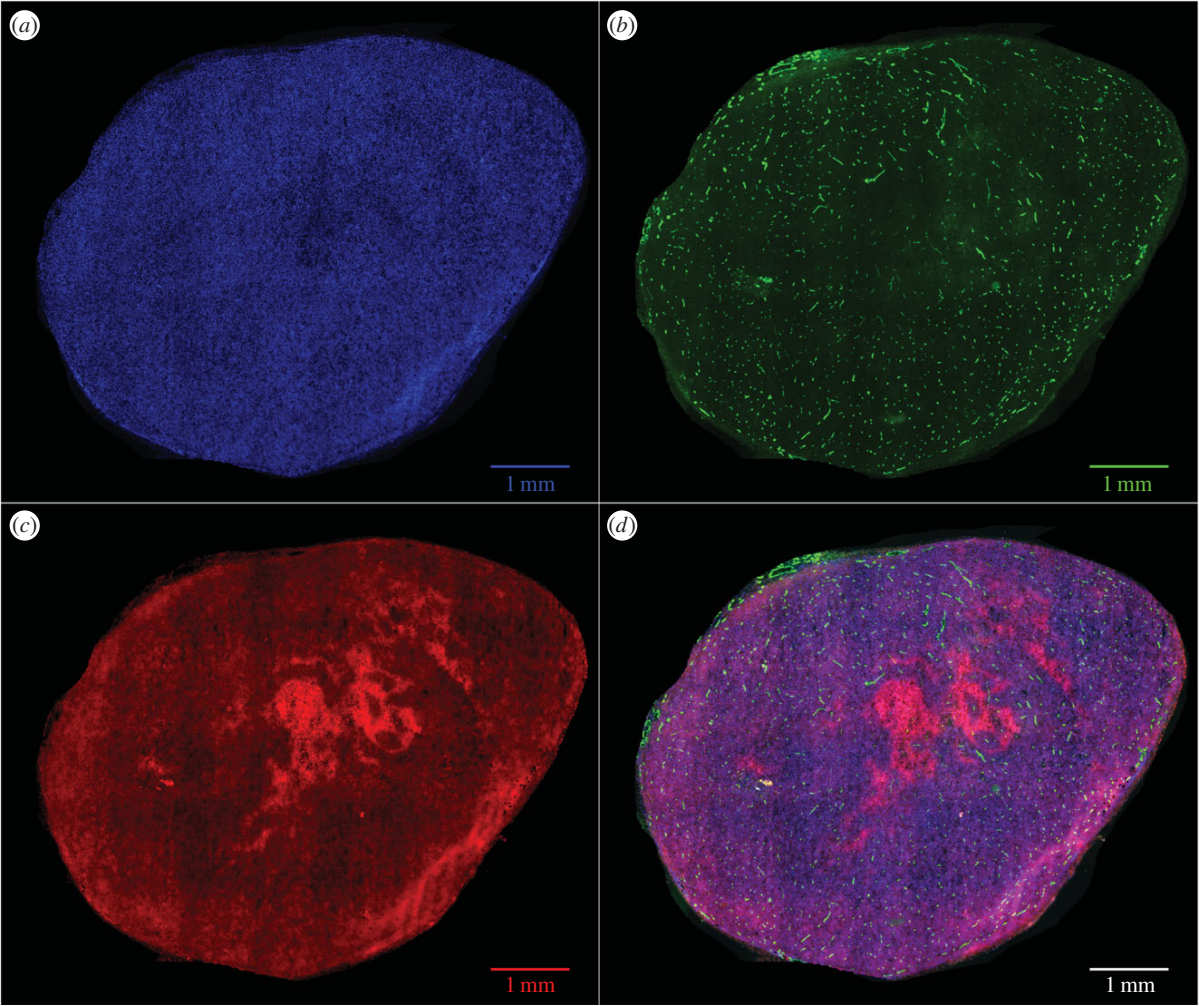
An MC-38 tumour, stained with (*a*) DAPI (blue), (*b*) vessel stain CD-31 (green), (*c*) hypoxia marker EF5 (red) and (*d*) all stained sections combined. Some regions of hypoxia are clearly visible in the light red regions, and more are apparent after image analysis.

With the EF5 channel extracted, a MATLAB script was used to create a mask, excluding areas of EF5 saturation at the tumour edge and null pixels with no tumour present. The adjacent sections with no EF5 were used as controls for each section analysed. The mean (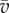) and standard deviation (*σ*) of pixels EF5 staining (i.e. red channel pixel value) was calculated across all pixels in the mask, and pixels with a value greater than 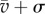 were taken as positive for EF5 staining. The fraction of EF5 staining was calculated by taking the total EF5 positive pixels over the total considered pixels in the tumour area. This yielded estimates of the fractions of the gross tumour at the EF5 binding oxygen tensions. This was compared with the simulated oxygen distribution through all slices through the MC-38 vessel section to investigate whether similar fractions would be obtained and the variability of these measurements. An example of an MC-38 tumour with all three stains visible is shown in figure 5. Sections with significant EF5 binding are clearly visible, and other regions with binding can be determined with the image analysis protocol outlined here.

#### 3.3.2. Theoretical analysis of two-dimensional vessel stains

The approach outlined in this work allows generation of a full three-dimensional oxygen map if three-dimensional architecture is known. For experimental reasons, histological analysis generally involves taking slices stained with a vessel marking agent such as CD31, highlighting endothelial cells in that image plane—these are shown in green in figure 5. A single slice will not necessarily convey information about adjacent slices, and might not be a suitable proxy for estimating the extent of oxygenation in tissue. To investigate how representative such slices are of the underlying three-dimensional vessel architecture, we simulated a histological vessel stain performed on a section of the mouse tumour by taking a slice of thickness Δ*W* around the centre of the vessel map. Vessels in the subsection are considered to lie in the same vertical plane, and two-dimensional analogue of the three-dimensional method is performed on the resultant grid. This analysis is performed with varying values of slice thickness Δ*W* and contrasted to a slice taken from the full three-dimensional kernel simulation to estimate how representative such histological slices might be.

## 4. Results

### 4.1. Three-dimensional oxygen maps

**Figure 6.**
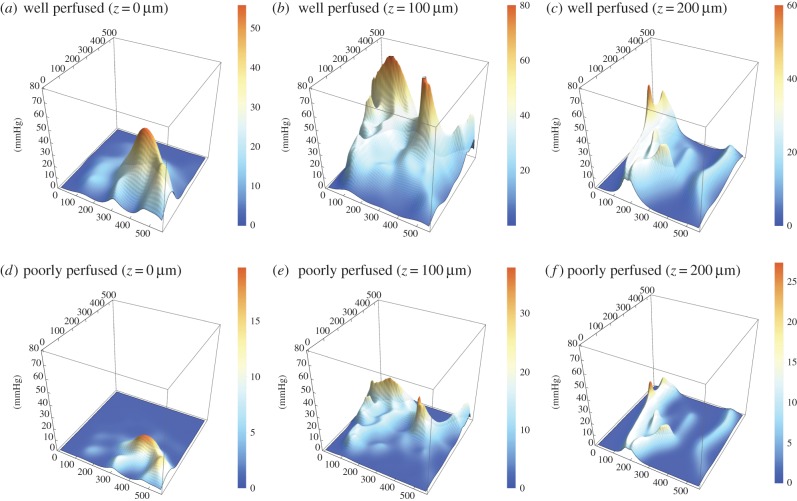
Contour maps for rat carcinoma as measured by Secomb *et al.* (*a*) Well perfused, *z* = 0 µm, (*b*) well perfused, *z* = 100 µm, (*c*) well perfused, *z* = 200 µm, (*d*) poorly perfused, *z* = 0 µm, (*e*) poorly perfused, *z* = 100 µm, (*f*) poorly perfused, *z* = 200 µm. Note that *z*-axes are constant between figures, but colour function differs between plots to facilitate comparison.

**Figure 7.**
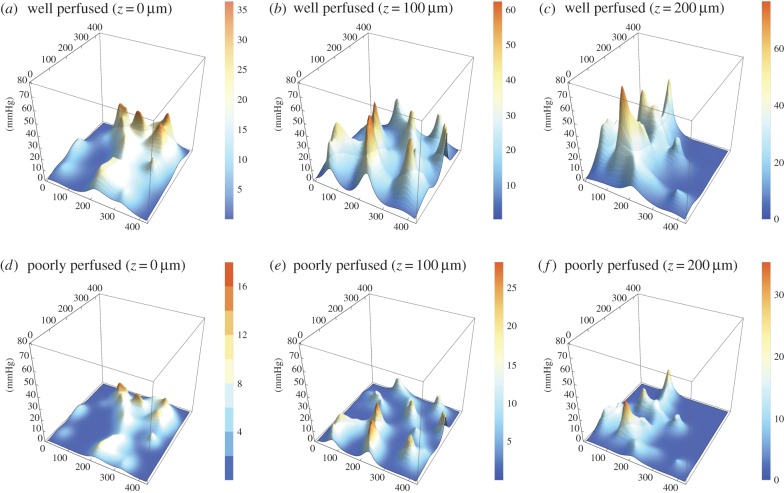
Contour maps for perfusion stained MC-38 mouse tumour grown for this work. (*a*) Well perfused, *z* = 0 µm, (*b*) well perfused, *z* = 100 µm, (*c*) well perfused, *z* = 200 µm, (*d*) poorly perfused, *z* = 0 µm, (*e*) poorly perfused, *z* = 100 µm, (*f*) poorly perfused, *z* = 200 µm. Note that *z*-axes are constant between figures, but colour function differs between plots to facilitate comparison.

For all three vessel maps, three-dimensional oxygen profiles were produced with the kernel method for both well and poorly perfused scenarios. Figure 6 illustrates the slices from the rat carcinoma illustrated in figure 3, under both assumptions of high and low perfusion. Similar maps for the low and high-density rat tumours are contained in the electronic supplementary material. Figure 7 shows the oxygen maps for the perfusion marked mouse tumour illustrated in figure 4. It is important to note that the murine tumour section only considers perfused vessels, whereas the rat carcinoma considers all vessels in the volume. Despite this, the hypoxic fractions are broadly similar between both sections, as can be seen in the hypoxia and anoxia statistics for these volumes which are given in table 2. The hypoxic and anoxic extents of both sections lie within 4% of each other under all conditions.

**Table 2.** Projected hypoxic and anoxic percentage for slices through the tumour volumes depicted in figures 3 and 4 under both well-perfused and poorly perfused assumptions.

section	perfusion status	hypoxia (%) (0 ≤ *p* ≤ 2.5 mmHg)	anoxia (%) (0 mmHg)
rat carcinoma	high	12.70	0.86
	low	42.07	7.27
mouse tumour	high	16.79	2.04
	low	45.61	8.15

### 4.2. Validation and comparison with two-dimensional histology

#### 4.2.1. EF5 staining results

Full sections were taken for the five MC38 mouse tumours and the percentage area per section stained with EF5 calculated. These tumour sections had an average area of 25 ± 8.7 mm^2^. The average EF5 staining in these sections was compared with the projected EF5 staining per vertical slice in the MC-38 section calculated using measured vessel information under the assumption of low and high perfusion, respectively. The results are shown in table 3. Agreement appears encouraging, though it is important to note that these sections had up to 100 times the area of the microvessel sections from the same mouse line, so the results should be cautiously interpreted. Further exploration of this is shown in the Discussion section.

**Table 3.** EF5 staining (two-dimensional versus three-dimensional comparison).

source	EF5-stained (%)
measured MC-38 sections	39.6 ± 9.1
estimated from MC-38 vascular network (high perfusion)	37.2 ± 9.8
estimated from MC-38 vascular network (low perfusion)	53.5 ± 13.2

#### 4.2.2. Two-dimensional vessel stain analysis

**Figure 8.**
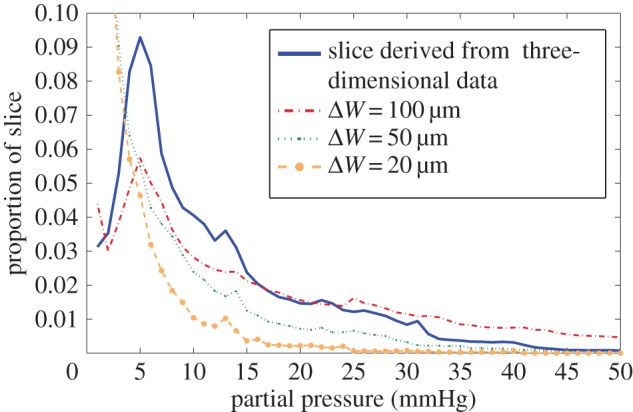
Oxygen histogram for a single slice of the MC-38 mouse tumour at *z* = 136 µm, derived from the full three-dimensional section and two-dimensional approximations created from vessel sections lying in the subvolume width Δ*W*. The oxygen histogram for two-dimensional slices does not in general represent the underlying distribution.

To investigate the implications of using a two-dimensional slice as a proxy for a true three-dimensional map, simulated cuts were taken symmetrically around the vertical centre of the rat mouse tumour at *z* = 136 µm. These cuts were respectively 100, 50 and 20 µm thick, and applied under the assumption of high perfusion, and contrasted with slices taken from the centre of the three-dimensional map. The relative oxygen distribution of these cuts is given in table 4. As can be seen from figure 8, the two-dimensional approximations do not represent the underlying three-dimensional map well; thick slices capture more vessels and because these are all assumed to be in the same vertical plane, it can give greater peak values and much higher mean O_2_ but also conversely overestimates hypoxia. With decreasing slice thickness, the two-dimensional approximation increasingly overestimates hypoxia. This heavily suggests that two-dimensional approximations will not suffice for representative oxygen distributions from complicated three-dimensional vasculature.

**Table 4.** Comparison of tissue oxygen status from histological cuts of various thickness with a slice from three-dimensional distribution.

slice	mean O_2_ (mmHg)	hypoxia (%)	anoxia (%)
three-dimensional cut	12.07 ± 3.36	6.66	0
100 µm two-dimensional cut	21.06 ± 6.70	7.39	0
50 µm two-dimensional cut	7.24 ± 4.45	40.43	0
20 µm two-dimensional cut	3.11 ± 2.33	62.88	2.02

## 5. Discussion

The kernel approach outlined here is powerful and straightforward to implement, and allows estimation of three-dimensional oxygen distribution with relative ease, taking into account intervessel variation. In this work, it has been applied to already existent vessel sections and a large section of mouse tumour, imaged using confocal microscopy to illustrate the applicability of the method. The model outlined in this work is useful for rapidly estimating oxygen distribution in three-dimensional space, but there are significant simplifications in this version of the model. First, the model expounded in this work neglects the longitudinal gradient in oxygen along the vessel, which can be significant even for the limited vessel lengths in this work; Vovenko [31] found that vessel partial pressure dropped an average of 17.9 mmHg from the arterial to venous end of long capillaries, with an estimated gradient of 0.07 ± 0.04 mmHg µm^−1^. For the longest vessel considered in this work (126 µm), this would constitute a change along the vessel length of Δ*P* = 8.82 ± 5.04 mmHg. This is however the extreme case; for the average vessel in the rat carcinoma (104 vessel sections), the projected O_2_ differences around the average vessel length would be Δ*P* = 5.03 ± 2.87 mmHg, and for the high-resolution mouse tumour (357 vessel sections) it would be only Δ*P* = 1.27 ± 0.72 mmHg. This variation is not presented in this work, but it could be readily extended to factor this in if flow vessels and direction can be ascertained.

It is important to note that there is significant experimental difficulty in determining which vessels are perfused and to what extent; one of the hallmarks of tumours are their chaotic and often poorly perfused vasculature [19]. To simulate this and show some of the range of possible situations, we applied the model under the assumptions of both well-perfused and poorly perfused conditions to give realistic upper and lower expected bounds for tissue oxygenation with a given vessel network. To isolate only perfused vessels, the mouse tumour was stained for vessels and perfusion, giving a clear image of active transporting oxygen. This analysis on this section yielded broadly similar values for the rat carcinoma as seen in table 2. Like all models, there are some simplifying assumptions made in this work—the oxygen consumption rate was taken as uniform throughout the tumour tissue in this work. In reality, this might perhaps vary with concentration of oxygen, yet recent work [12] suggests that even if oxygen consumption rate decreased in a hyperbolic fashion with available oxygen the kernel would differ from the uniform consumption case only by a negligible amount. The oxygen consumed at any given point may come from multiple vessels, so there is no obvious *a priori* way to explicitly assign a specific consumption rate in tissue per unit length of the vessel in these circumstances, but the rapid fall-off of oxygen contribution from a point source (illustrated in figure 2*a*) leads us to the simplification that all points in three-dimensional space can be independently considered. For these reasons, uniform consumption appears a reasonable assumption in most cases.

A benefit of the described kernel method is that it allows arbitrarily high-resolution oxygen maps to be predicted in a three-dimensional tissue volume in a computationally efficient manner. With this approach, an experimentally measured diffusion distance *r_n_* allows one to calculate the estimated oxygen maps of any three-dimensional volume. Thus, the kernel method is parametrized by a measurable parameter. By contrast, the bulk tissue consumption rate is a necessary input to the numerical method which is impossible to determine directly from histology therefore multiple simulations must be carried out to fit this parameter, each of which comes with a computational cost. As a result, numerical calculations are typically performed on much lower resolution grids, a notable exception being the approach of Secomb *et al.* [24]. Nevertheless, it is important to compare the method outlined here with conventional numerical methods to identify differences between approaches.

A full comparison of the kernel method with a finite difference method is provided in the electronic supplementary material for both isolated vessels and a known three-dimensional vessel network. In summary, the approaches agreed for isolated vessels with a r.m.s error of less than 0.2 mmHg. In the three-dimensional vessel network, broad agreement was seen between both approaches; the major features of the 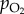 distributions were similar; however, a larger saturated area of oxygenation was seen in the case of the kernel model, and the finite difference solution displayed shallower fall-off. These differences may be attributed to three factors. These were specifically the different implementations of consumption, the different resolutions of the calculation grids and potential overestimation from overlapping vessels in the kernel model. The larger saturated area is unlikely to have implications for radiotherapy, as maximum OER is achieved at partial pressures beyond 20 mmHg [4] after which no further dose boosting effects are seen. From a radiobiological perspective, the shallower fall off in the numerical model (itself determined by consumption rate chosen) might be of more interest, as dose boosting effects are at their most stark less than or equal to 3 mmHg oxygen pressure. However, for the vessel map shown in electronic supplementary material, S1(d), this would not have any significant effects on dose prescription between models. This is an avenue for future exploration.

The method here was employed not only to estimate oxygen distribution in three-dimensional vessel sections, but also to simulate histological sampling and estimate the type of errors ignorance of the three-dimensional situation might lead to. This is examined in table 3; with thicker slices, more blood vessels can be seen in the staining plane. If these are assumed to lie in the same vertical domain, it can lead to overestimations of the mean oxygen partial pressures, but the loss of the underlying three-dimensional information also means such a simplification is liable to overestimate the extent of hypoxia. With smaller slices, the mean value estimates decrease rapidly and so too does the overestimation of hypoxia. Figure 8 illustrates the histograms for these different sections, clearly illustrating that none of the two-dimensional approximations closely match that derived from the three-dimensional architecture. This implies that simple two-dimensional histological data are not adequate to give an acceptable approximation of the true three-dimensional case, strongly suggesting that there is no substitute for accurate three-dimensional vessel maps if reliable oxygen estimates are to be obtained. While the two-dimensional simulations were compared with the three-dimensional model derived here, the discrepancy between two-dimensional histology and three-dimensional supply with occur regardless of the models used as vessels outside the slice plane will always act to distort estimates.

There is another interesting conclusion that might follow from this; if oxygen at a given point *K* is to be estimated, then full knowledge of the surrounding vasculature is vital. The spatial extent of this domain required can be readily shown to simply be the maximum projected diffusion limit *r_f_*, so that the sphere of influence around *K* has radius *r_f_* to be free from confounding influences from other vessels. Thus, to completely estimate the oxygen distribution inside a cuboid of volume *XYZ* would require full vessel architecture information from a greater volume *V*, given by 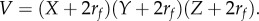 For the sections, in this work, this implies that vessels with typical diffusion distances of between 100 and 150 µm outside the subvolumes might influence the projected oxygen map, decreasing projected hypoxia. Because of the rapid drop off of oxygen with diffusion distance, as illustrated by the kernel in figure 2*a*, this effect should only manifest at the volume edges leaving the centre projection relatively consistent.

While the method and analysis outlined in this work are broadly applicable, the heterogeneous nature of the tumour microenvironment means that both nutrient supply and vascular architecture can vary markedly in space and time. With knowledge of the vessel architecture in a tumour subvolume, the method outlined here (or other similar approaches, such as numerical or Green's function methods) may yield accurate estimation of the local tumour oxygenation around that network, but a sampled section of tumour cannot be directly extrapolated to yield an oxygen distribution for an entire tumour. Similarly, two-dimensional histology in isolation is generally inadequate for determining oxygen distribution throughout the tumour, as explored in this work. This also means that the MC-38 tumour sections analysed with EF5 must be interpreted very cautiously; these are taken from large two-dimensional histological sections, and while it is encouraging that average oxygen distributions seem similar to those taken from a three-dimensional section with perfusion markers (table 3), this in itself should not be considered a validation owing to the potential for heterogeneity in tumours. Full three-dimensional vessel information for the entirety of a large tumour remains experimentally challenging to obtain, and to the best of authors' knowledge is not yet technically feasible. The work here should be considered a proof of principle, outlining the importance of three-dimensional oxygen distribution and a rapid method for estimating it when vessel structure and perfusion is known.

Knowledge of the regional oxygenation status within a tumour has great potential benefits in radiotherapy, because it has long been known that well-oxygenated tumours respond better to radiation treatment by up to a factor of 3 relative to anoxic regions [3]. This oxygen enhancement effect is likely a direct consequence of oxygen radical products [4] and, in principle, areas of low oxygen could be targeted with an increased dose to overcome the increased radioresistance associated with hypoxia. Quantification of the spatial and temporal distributions of oxygen in clinical practice, for example using ^18^F-fluoromisonidazole PET, is limited by the physics of the imaging system to the millimetre regime, making estimation of dose–response on a cellular level very challenging. Tumour oxygenation also varies dynamically, with both transient microscopic changes being a feature of bulk hypoxic regions, and in response to treatment through angiogenesis and/or changes in oxygen consumption. Vessel modelling cannot directly alleviate these problems, but mathematical simulations from measured three-dimensional vasculature give insights into macroscopic oxygen levels that would be observed from various classes of underlying vessel microstructure, for example studying vascular remodelling in response to treatment in preclinical models [32].

Validating three-dimensional oxygen maps at the vessel length-scale remains technically difficult; even measuring three-dimensional perfused vasculature maps as outlined in this work is experimentally challenging. It was not technically feasible for us to measure spatial hypoxia distribution and three-dimensional vessel architecture in the same tumour, so a study was performed comparing the experimentally measured (EF5-stained) and mathematically modelled hypoxic fraction derived from a measured three-dimensional perfused vessel map in the same cohort of mice with identically treated subjects. Although the agreement is encouraging, it is important to note that directly comparing the average distributions of EF5 sections at the millimetre length-scale with those derived from micrometre maps is only valid under the assumption that average oxygen distributions remain similar with scale; the mouse sections resolvable with EF5 were approximately 100 times the area of those sections with imaged microvessels, corresponding to approximately 10 times the length-scale in each direction. Whether the assumption of approximate consistency over this distance is fully justified is not currently known, and simultaneous spatial measurements of hypoxia and vasculature within the same tumour would provide a more definitive validation.

## 6. Conclusion

This work establishes a novel and powerful analytical model for rapidly estimating oxygen distribution in three dimensions from vessel architecture, and demonstrates how it can be applied to vessel segments derived from confocal microscopy with perfusion markers. Two-dimensional histological slices are also contrasted to full three-dimensional distributions, with results suggesting that two-dimensional sections are not suitable for the complexities of three-dimensional oxygen estimation in tumours. Despite the fundamental importance of tumour hypoxia, direct estimation of microenvironment oxygenation is still technically challenging, and further research is needed to quantify this fully and use this information in oncological interventions.

## Supplementary Material

S1: Mathematical Appendices
