# Supplementary material for: Estimating oxygen distribution from vasculature in three-dimensional tumour tissue
Source: J R Soc Interface. 2016 Mar;13(116):20160070. doi: 10.1098/rsif.2016.0070 (PMC4843681; doi:10.1098/rsif.2016.0070)
Supplement: S1: Mathematical Appendices [file rsif20160070supp1.pdf]

Estimating oxygen distribution from vasculature in three-dimensional tumor tissue - Mathematical Appendices

David Robert Grimes[1,*], Pavitra Kannan[1], Daniel R Warren[1], Bostjan Markelc[1], Russell Bates[2], Ruth Muschell[1], Mike Partridge[1]

[1] Cancer Research UK/MRC Oxford Institute for Radiation Oncology, Gray Laboratory, University of Oxford, Old Road Campus Research Building, Off Roosevelt Drive, Oxford OX3 7DQ, UK

[2] Engineering Sciences, University of Oxford, OX1 3PJ, UK

[*] E-mail: davidrobert.grimes@oncology.ox.ac.uk

Appendix A - Derivation of spherical point source

If we consider a spherical point source diffusing oxygen that is consumed at an approximately uniform rate a, we can describe the oxygen diffusion from this point source P_I with a reaction-diffusion equation given by

$$\frac{\partial P_I}{\partial t} = D\Delta P_I - a\Omega s_L = \frac{D}{r^2}\frac{\partial}{\partial r}\left(r^2\frac{\partial P_I}{\partial r}\right) - a\Omega s_L \tag{1}$$

where s_L is a scaling constant. We further assume that the oxygen quickly reaches a steady-state distribution, so we can let $\frac{\partial P_I}{\partial t} = 0$. This reduces the equation to a second-order homogeneous linear equation with variable coefficients. From the point source, the oxygen is steadily consumed until traveling a radial distance of r_n with zero flux at the boundary, so the boundary conditions are

$$\left.\frac{\partial P_I}{\partial r}\right|_{r_n} = P_I(r_n) = 0 \tag{2}$$

This is analytically tractable, yielding the solution

$$P_I = \frac{a\Omega s_L}{6D}\left(r(z)^2 + \frac{2r_n^3}{r(z)} - 3r_n^2\right). \tag{3}$$

P_I itself is the output from a point source along an orthogonal line element dz, so we may rewrite this is terms of total oxygen contribution from a line source P by $P_i = \frac{dP}{dz}$. This yields the first equation in the main-text, which can be integrated along z as described in the article.

Appendix B - Segment kernel points

To place kernels along a vessel, segments must first be discretised. For a segment with length L microns and endpoints $E_0 = \begin{pmatrix} x_0 \\ y_0 \\ z_0 \end{pmatrix}$ and $E_N = \begin{pmatrix} x_N \\ y_N \\ z_N \end{pmatrix}$, the respective vessel angles in the XY plane θ and XZ plane ϕ were respectively given by

$$\theta = \arctan\left(\frac{y_N - y_0}{x_N - x_0}\right) \tag{4}$$

$$\phi = \arctan\left(\frac{z_N - z_0}{x_N - x_0}\right). \tag{5}$$

The effective length in the XZ and XZ planes was then given by $R_{XY} = \sqrt{(x_N - x_0)^2 + (y_N - y_0)^2}$ and $R_{XZ} = \sqrt{(x_N - x_0)^2 + (z_N - z_0)^2}$ respectively. For a given vessel segment, the incremental positions of the i discrete kernel points from

$$\begin{pmatrix} x_i \\ y_i \\ z_i \end{pmatrix} = E_0 + \begin{pmatrix} \frac{L}{R_{XY}}(i)\cos(\theta) \\ \frac{L}{R_{XY}}(i)\sin(\theta) \\ \frac{L}{R_{XZ}}(i)\sin(\phi) \end{pmatrix} \tag{6}$$

Appendix C - Perpendicular distance in 3-Space

For accurate normalization in 3D space, the vector equation of the segment line is given by $E_n - E_0$, yielding the matrix

$$\begin{pmatrix} A \\ B \\ C \end{pmatrix} = \begin{pmatrix} x_N - x_0 \\ y_N - y_0 \\ z_N - z_0 \end{pmatrix}. \tag{7}$$

For a line in 3-space, we may define the polar and azimuth angle respectively as

$$\theta = \arctan\left(\frac{B}{A}\right) \tag{8}$$

$$\varphi = \arccos\left(\frac{C}{\sqrt{A^2 + B^2 + C^2}}\right). \tag{9}$$

If the centre of this vessel segment is at the point C, the co-ordinates of a point a perpendicular distance of r_f from C and the segment can be found by manipulating the spherical co-ordinate identities, which yields an expression for the co-ordinates of a perpendicular normalization point W given by

$$\begin{pmatrix} W_x \\ W_y \\ W_z \end{pmatrix} = C + r_f \begin{pmatrix} \cos\theta\cos\varphi \\ \cos\theta\sin\varphi \\ -\sin\theta \end{pmatrix}. \tag{10}$$

Provided care is taken to ascertain the correct quadrant for the arctangent, the algorithm above will find a perpendicular point a distance r_f from the centre of any line in three-space.

Appendix D - Numerical Methods

Oxygen distributions calculated by the kernel convolution method were compared to the those predicted by numerical methods. For this comparison we developed a MATLAB program, which calculates approximate steady-state solutions to the diffusion equation using the finite difference method on a three-dimensional Cartesian grid. The fixed boundary condition $p = p_0$ is imposed at the surface of vessels, which are represented by rasterized cylinders. The fixed boundary condition $p = 0$ is imposed at the outer edges of the domain, which are located at a distance greater than r_n from the nearest vessel. It is assumed that steady state is reached when volume-averaged $\frac{\mathrm{d}p}{\mathrm{d}t} < 10^{-6}$ mmHg.

Radial oxygen profiles around vessels were also compared against limiting-case spherical and cylindrical geometries calculated at high-resolution using the MATLAB function `pdepe`. Both numerical methods solve the following equation, which models the oxygen consumption process using a bulk tissue consumption rate q in place of the $a\Omega$ term employed by the kernel method

$$\frac{\partial p}{\partial t} = D\nabla^2 p - q$$

Figure 1 shows the predictions of each method for radial p_{O_2} profiles around vessel segments with 10μm diameter and lengths of 10μm, 100μm and 300μm. For each numerical simulation, a value of q was found that produces a profile consistent with $r_n = 150\mu$m. In the finite difference simulations, 10μm vessels required $q = 0.05$ mmHg/s, 100μm vessels required $q = 0.86$ mmHg/s, and 300μm vessels required $q = 1.73$ mmHg/s. The one-dimensional simulations with MATLAB's `pdepe` function used a tissue consumption rate of 0.05 mmHg/s for 10μm diameter spherical vessels, and a rate of 2.2 mmHg/s for infinite cylindrical vessels.. The predictions of the kernel model and the finite difference model show good agreement with each other (r.m.s. error < 0.2 mmHg) and lie within the range that would be expected, given the results of the geometrically-simple limiting cases.

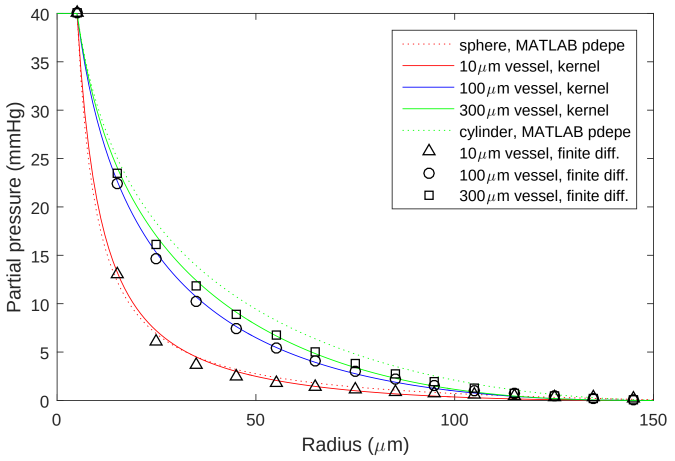

Figure 1: Comparison of three methods for calculating radial oxygen profiles around a vessel segment. The kernel model and a finite difference method are used to model 10μm diameter cylindrical vessel segments with lengths 10-300μm. Results are additionally shown for spherical and infinitely-long cylindrical vessels, calculated using MATLAB's one-dimensional PDE solver `pdepe`.

Further comparison was made between the kernel model and finite difference method solutions using a 3D vessel network, from data published by Secomb et. al [1] shows the 28-segment network, which is specified in a tissue volume measuring $335\mu m \times 225\mu m \times 205\mu m$ as shown in figure 2.

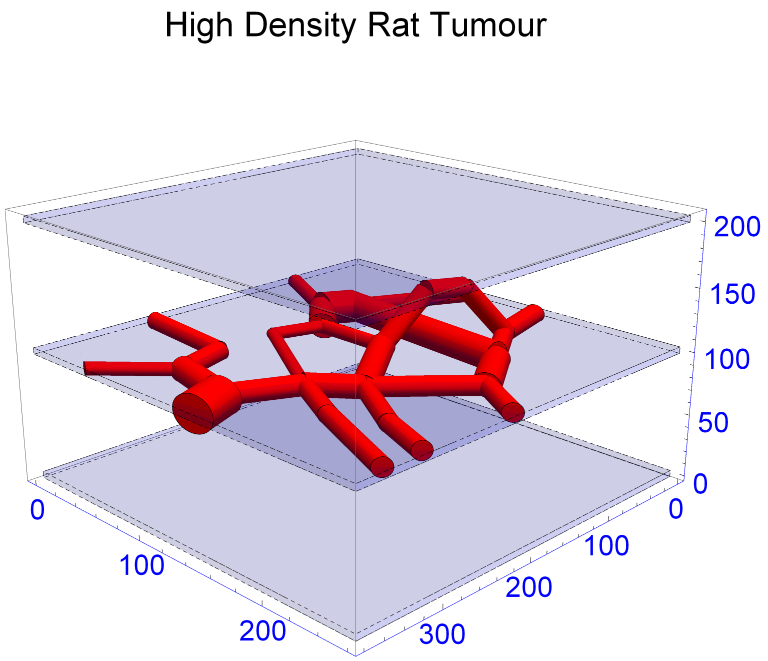

Figure 2: A high density rat carcinoma vessel map taken from Secomb et al [1]

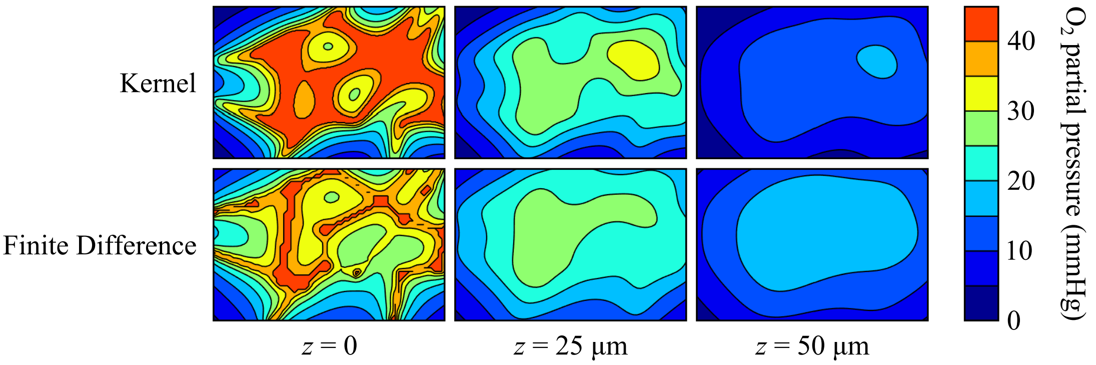

Figure 3: Comparison of oxygen distributions calculated using the kernel model and a finite difference method. Results are shown for three slices within a 3D vessel network shown in figure 2. The coordinate z is measured from the central plane of the network, in which most of the vessels lie.

The kernel solution at each point comprises a sum of contributions from each vessel's radial profile with $r_n = 150\mu$m calculated at 1μm resolution. The total sum was scaled such that mean vessel surface p_{O_2} is p_0, and oxygen partial pressure within vessels was set to the same value. Finite difference solutions were calculated using a 3D map of the vessel network rasterized at 5μm resolution and assumed a tissue consumption rate of 1.73 mmHg/s. Contour plots of slices through the oxygen distributions determined by each method are shown in figure 3.

Both methods show broad agreement in the location and shape of high-and low-p_{O_2} features surrounding this complex vessel network. It is notable that the area of full oxygen saturation in the vessel plane is considerably larger in the case of the kernel model. A direct sum of kernels may overestimate the oxygen influx near the surface of vessels located in close proximity, because the concentration gradient is likely to be shallower than assumed in the kernel derivation. Alternative techniques for combining kernels from multiple vessels may improve predictions in the case of vessel networks, and will be the subject of future modelling work. Some of the differences seen may be explained by the fact that the finite difference calculations have been performed at considerably lower resolution. This will introduce a degree of error in the shape and size of the vasculature, and there will be loss of detail in the calculated maps due to volume-averaging effects. The finite-difference model also makes an additional assumption of constant oxygen consumption rate, whilst this parameter may vary in the kernel model in order to ensure a fixed value of r_n. The effect is apparent at larger distances from vessels, where oxygen distributions extend further in the case of the finite difference model.

References

[1] Secomb TW, Hsu R, Dewhirst MW, Klitzman B, Gross JF. Analysis of oxygen transport to tumour tissue by microvascular networks. International Journal of Radiation Oncology*Biology*Physics. 1993;25(3):481 – 489.

[2] Hall E, Giaccia A. Radiobiology for the radiologist (6th Edition). Lippincott William and Wilkins, Philadelphia; 2006.

[3] Grimes DR, Partridge M. A mechanistic investigation of the oxygen fixation hypothesis and oxygen enhancement ratio. Biomedical Physics & Engineering Express. 2015;1(4): 045209.
